# Diagnosis of *Apis dorsata* venom allergy: use of recombinant allergens of *Apis mellifera* and a passive basophil activation test

**DOI:** 10.1186/s12948-022-00178-9

**Published:** 2022-09-14

**Authors:** Peshala Gunasekara, S. M. Handunnetti, Sunil Premawansa, E. W. R. A. Witharana, Indra P. Ratnayake, Pradeep Kaluarachchi, Chandima Karunatilake, R. K. S. Dias, G. A. S. Premakumara, W. M. D. K. Dasanayake, Suranjith L. Seneviratne, Rajiva de Silva

**Affiliations:** 1 Institute of Biochemistry, Molecular Biology and Biotechnology, University of Colombo, Colombo 3, Sri Lanka; 2 Department of Zoology and Environment Sciences, Faculty of Science, University of Colombo, Colombo 3, Sri Lanka; 3 Base Hospital, Deniyaya, Sri Lanka; 4 District Hospital, Bandarawela, Sri Lanka; 5 A Baur & Co. Pvt. Ltd, Healthcare Division, No. 62, Jethawana Road, Colombo 14, Sri Lanka; 6 Department of Immunology, Medical Research Institute, Colombo 8, Sri Lanka; 7 Department of Zoology and Environmental Management, Faculty of Science, University of Kelaniya, Dalugama, Sri Lanka; 8 Department of Basic Sciences & Social Science, University of Colombo, Colombo, Sri Lanka; 9 Institute of Immunity and Transplantation, Royal Free Hospital and University College London, London, UK; 10 Department of Surgery, Faculty of Medicine, University of Colombo, Colombo 8, Sri Lanka

**Keywords:** *Apis dorsata*, *Apis mellifera*, Recombinant allergens, CD63, Cross-reactivity

## Abstract

**Background:**

Allergy to *Apis dorsata* (Giant Asian Honeybee) venom is the commonest insect allergy in Sri Lanka and South East Asia. However, laboratory diagnosis is difficult as the pure venom and diagnostic reagents are not commercially available.

**Objective:**

This study assessed the use of four recombinant allergens of *A. mellifera* venom and the passive basophil activation test in the diagnosis of *A. dorsata* venom anaphylaxis.

**Methods:**

Serum IgE levels to four recombinant allergens of *A. mellifera*, rApi m 1, 2, 5 and 10 were assessed and compared with serum IgE to the crude venom of *A. mellifera* or *V. vulgaris* by Phadia ImmunoCAP, in patients who developed anaphylaxis to *A. dorsata* stings. Basophil activation in response to venom of *A. dorsata* or *V. affinis* was assessed using a passive basophil activation test. Association of the severity of the reaction with basophil activation was compared.

**Results:**

rApi m 1 and 10 combinedly had significant correlation (r = 0.722; p < 0.001) with the crude venom of *A. mellifera* (Western honeybee) and a higher positivity rate of 90% (27/30). Whereas, IgE reactivity to rApi m 2 or 5 had significant correlation (p = 0.02 and p = 0.005 respectively) with *V. vulgaris* crude venom. All 30 (100%) were positive to *A. dorsata* venom in passive BAT; 70% (21/30) had over 80% activation, 96.7% (29/30) had over 60% activation and 100% had over 50% activation. Percentage activation of basophils in patients who had mild or moderate reactions (n = 20) was significantly low (p = 0.02) from that of patients who had severe reactions (n = 10).

**Conclusions:**

rApi m 1 and 10 when combined was sensitive for the diagnosis of *A. dorsata* allergy. This combination had the lowest cross-reactivity rate with *Vespula vulgaris*. The passive BAT is highly sensitive in *A. dorsata* allergy. The basophil reactivity was significantly higher in severe anaphylaxis compared to mild/moderate anaphylaxis. This finding should be further explored in further studies.

## Introduction

*Apis dorsata* (Giant Asian Honeybee) is restrained to a small geographical area (South East Asian region) compared to the Western honeybee, *A. mellifera* [1, 2]. Allergy to *A. dorsata* venom is the commonest venom allergy in Sri Lanka and South East Asia [3–5]. A study carried out in Deniyaya (Southern Province of Sri Lanka) using 322 Hymenoptera (*A. dorsata* and *Vespa* ssp) venom allergic patients from September 2011 to August 2013 [3], revealed that 90.7% (292) were due to *A. dorsata* stings. An epidemiological study [6] in the North Western Province of Sri Lanka in 2018 noted 357 cases of Hymenoptera (*A. dorsata* and *V. affinis*) sting allergies. Of those, 92% were from Primary Care Hospitals (PCHs) and only 8% were from Tertiary Care Hospitals (TCHs). The study also identified underreporting of symptoms and adverse reactions, mainly systemic reactions, in Hymenoptera sting allergy [6]. Therefore, many cases of sting related allergies remain unnoticed. Anaphylaxis following Hymenoptera stings (*A. dorsata* and *Vespa* ssp) was 4.6% (15/322) in the study from Deniyaya, Sri Lanka [3], whilst a higher percentage (30%, 110/357) of systemic reactions was noted in the study carried out in the North Western Province. Fatalities due to *A. dorsata* venom allergy are reported in Sri Lanka [4, 6], Thailand [7], Pakistan [8], India [9, 10] and Nepal [11–13]. However, despite the fact that allergies due to *A. dorsata* venom are common, diagnostic grade venom preparations of *A. dorsata* are not commercially available. Our previous studies [4] have shown the similarity of venom of *A. dorsata* with that of *A. mellifera*, and the possible cross-reactivity of phospholipase A_2_ (PLA_2_) and hyaluronidase of *A. dorsata* and *A. mellifera* venom. This cross-reactivity indicated the possibility of using *A. mellifera* venom/components in the diagnosis of *A. dorsata* venom allergy until the diagnostic grade venom preparations of *A. dorsata* are commercially available.

Venom specific IgE (sIgE) detection using in vivo (skin prick and intra dermal tests) or in vitro (i.e. Phadia ImmunoCAP) tests are widely used in the diagnosis of Hymenoptera insect venom allergies [14–16]. However, false-positive results may be observed in sIgE detection by in vitro methods due to cross-reactive carbohydrate determinants (CCD) present in natural venom proteins of bees and wasps [17]. Therefore, component resolved diagnosis (CRD) has been developed to detect sIgE using CCD-free individual venom proteins from bees and wasps [18–21]. Recombinant PLA_2_ (rApi m 1) was the first allergen of the Western Honeybee used in CRD. However, due to the low sensitivity (ranging from 58 to 80%) [22–25] associated with rApi m 1, the following recombinants proteins; hyaluronidase (rApi m 2), dipeptidyl peptidase IV (rApi m 5) and icarapin (rApi m 10) were manufactured using *Spodoptera frugiperda* (Sf9) insect cell-based expression system [26]. These allergens are now being used in CRD to detect honeybee venom (HBV) allergy. The reported IgE reactivity to these allergens was lower than Api m 1 (47. 9%—rApi m 2, 58.3%—rApi m 5 and 61.8%—rApi m 10) [21]. However, a study conducted with a panel of five recombinant allergens (rApi m 1, 2, 4, 5 and 10) has shown increased sensitivity (94.4%) [21].

The conventional basophil activation test (BAT) may be useful in the diagnosis of *A. dorsata* venom allergy as basophils have surface markers that are upregulated following cross-linking of surface IgE [27]. The patients’ basophils probably coated with allergen-specific IgE on its surface, are exposed to the responsible venom resulting in activation of the basophil. Activated basophils express activation markers (CD63 or CD203c) on its surface which can be measured using flow cytometry. However, one of the major downfalls of BAT is that the analysis needs to be performed within 4 h of blood collection [27]. This is an issue in countries such as Sri Lanka where most of the patients are from remote areas. Two studies had shown that basophils from one individual could have their surface IgE replaced by IgE from a different donor [28, 29]. We have recently evaluated the use of a passive BAT in diagnosis of *V. affinis* (a hornet species which is native to Sri Lanka) [30]. However, the passive BAT concept has still not been tested in honeybee venom allergies. Thus, we tried to produce a passive BAT using basophils from healthy donor whose membrane-bound IgE have been removed using a mild acidic condition and passively sensitized with IgE from *A. dorsata* venom allergic patients. With adequate activation, the passive BAT could be considered as a useful diagnostic test for *A. dorsata* venom allergy.

In the present study, we used four commercially available recombinant allergens of *A. mellifera* in the diagnosis of *A. dorsata* venom allergy. In addition, we assessed the possibility of using membrane-bound IgE depleted healthy donor basophils. These basophils were then tested for activation following the addition of serum from patients who developed anaphylaxis to *A. dorsata*, a passive BAT. The analyses were prospective and blinded. If a higher percentage of activation could be determined compared to the Phadia ImmunoCAP, then the passive BAT could be useful in the diagnosis of *A. dorsata* venom allergy, and potentially, allergy in other instances as well.

## Methodology

### Ethical statement

Ethics approval for this study was obtained from the Ethics Review Committee, Medical Research Institute, Colombo, Sri Lanka (No: 46/2013).

### Patients

The study group consisted of 30 confirmed *A. dorsata* venom anaphylaxis patients (20 males and 10 females with an age range of 16–83) from Deniyaya Base Hospital (n = 13) and Bandarawela District Hospital (n = 17), Sri Lanka. Serum from each patient was collected after obtained written consent from each patient. All patients were shown a panel of dead specimens of various Hymenoptera stinging insects to confirm the insect responsible for stinging. Patients who identified the responsible insect as *A. dorsata* and who had no previous allergies to other Hymenoptera insects were selected. Through a questionnaire, relevant clinical history (Table 1) of each patient was gathered and characterized according to the British Society of Allergy and Clinical Immunology (BSACI) guidelines; Mild- pruritus, urticaria, erythema, mild angio-oedema, rhinitis, conjunctivitis, Moderate- mild asthma, moderate angio-oedema, abdominal pain, vomiting, diarrhoea, minor and transient hypotensive symptoms (light headedness, dizziness) and Severe- respiratory difficulty (asthma/laryngeal oedema), hypotension, collapse or loss of consciousness, incontinence, seizures, loss of colour vision [31]. Patients were divided into two groups based on the severity of the anaphylaxis reaction: group 1—mild or moderate anaphylaxis (n = 10) and group 2—severe anaphylaxis (n = 20).

**Table 1:** Clinical data of the selected patients

Clinical reaction	Total and percentage (%; n = 30)
Mild	
Pruritus	15 (50%)
Urticaria	16 (53%)
Erythema	–
Mild angio-oedema	30 (100%)
Rhinitis	–
Conjunctivitis	–
Moderate	
Mild asthma	7 (23%)
Moderate angio-oedema	8 (26%)
Abdominal pain, vomiting	8 (26%)
Diarrhoea	5 (17%)
Minor and transient hypotensive symptoms (light headedness, dizziness)	4 (13%)
Severe	
Respiratory difficulty (asthma/laryngeal oedema)	–
Hypotension	10 (33%)
Collapse or loss of consciousness	20 (66%)
Incontinence	4 (13%)
Seizures	–
Loss of colour vision	–

### Detection of IgE reactivity

Venom specific IgE reactivity was detected using ImmunoCAP i1 containing crude venom of *A. mellifera* (immunologically similar to *A. dorsata*) and ImmunoCAP i3 containing *V. vulgaris* crude venom. Phadia ImmunoCAP of rApi m 1, 2, 5 and 10 were purchased and IgE reactivity was detected using the Phadia 100 system. The cutoff was set as > 0.35 kU_A_/L for sIgE positivity. The patients who had positive IgE to both *A. mellifera* and *V. vulgaris* crude venom were considered as double-positives. The samples which had double-positivity were pooled into three categories according to the IgE positivity to *V. vulgaris* (wasp) venom; Category 1—low IgE (0.35–3.4 KU_A_/ L), Category 2—moderate IgE (3.5–17.4 KU_A_/ L) and Category 3—high IgE (17.5–50 KU_A_/ L). IgE quantity to *A. mellifera* venom and its components in two severity groups were compared.

### In vitro sensitization of basophils from healthy donors

Passively immune basophils were prepared according to a method developed and used by us [30] and others [32]. An aliquot of 100 µL heparinized whole blood from a healthy donor was used in each test sample. IgE in donor basophils was removed as described in our previous study [30]. Briefly, using 500 µL of Sodium lactate solution (14 mM Sodium lactate, 145 mM of NaCl and 6 mM KCl), each basophil sample was incubated for 15 min. The blood samples were centrifuged (1200*g* for 5 min) at 4 °C. The supernatant was discarded and the pellet was resuspended in 100 µL of 0.13 M PBS. Afterward, the samples were incubated with 50 µL of serum of either *A. dorsata* venom allergic patients or patients having different allergies (IgE control sera) for 60 min at 25 °C.

### Passive basophil activation test using patient serum

The passive BAT was carried out using the sera from 30 patients selected. Two positive controls were used; polyclonal anti-IgE (Sigma Aldrich) at 1:100 dilution and fMLP provided with the kit (100 µL of 0.002 mM/ mL). Two sets of negative controls were used in the test, i.e., (i) donor basophil samples (without replacing IgE) were incubated with 100 µL of the wash buffer and (ii) IgE depleted donor basophils were incubated with 100 µL of pooled control sera from 7 patients who were not allergic to hymenopteran insects. Gunasekara et al. [30] and others [27, 33–35] established that 1 µg/mL was the most suitable concentration of Hymenoptera venom to activate the basophils. Hence, 1 µg/mL of *A. dorsata* venom was used in this study. Following incubation, 1 µg/mL of *A. dorsata* venom was added to each tube comprising serum and was incubated for 20 min at 37 °C. Activated basophils were stained for 20 min using a 20 µL mixture of anti-IgE-PE and anti-CD63-FITC provided with the kit on an ice bath.

Lymphocyte/basophil fraction was selected by gating side scatter vs forward scatter. Basophils were selected using the side scatter low-IgE high cell population. The tests were analyzed acquiring at least 200 basophils using FACSCalibur flow cytometer (BD Bioscience) [27]. The percentage activation of CD63 (basophil population anti-CD63-FITC intensity > 10^2^) was then calculated from the gated population. The cutoff point in passive BAT was set as mean of negative controls + 3.3 SD [30, 36]. In addition, the percentage basophil activation in response to *A. dorsata* venom in the two severity groups was compared. Three categories of double-positives (by Phadia ImmunoCAP) were tested using the passive BAT.

### Determination of donor dependency

Basophils from a second donor were used to determine the donor dependency of the test. Two tests were repeated using those basophils; sera of two patients (patient# 5 and 10) were incubated with the IgE depleted donor basophils. These were then incubated with *A, dorsata* venom (1 µg/mL) and percentage CD63 activation were determined.

### Statistical analyses

SPSS package (IBM, version 20) and Prism 8.0 (GraphPad, California, USA) were used for the statistical analyses. Non parametric tests were used since the data was not normally distributed. Possible correlations in IgE positivity to *A. mellifera* (or venom components) with the crude venom of *V. vulgaris* were determined using Spearman correlation. IgE positivity to crude venom of *A. dorsata* in the two severity groups in the passive BAT: group 1 (mild + moderate) and group 2 (severe) was compared using Mann–Whitney U test. Values of P < 0.05 at 95% confidence level were considered significant.

## Results

### Determination of venom specific IgE

**Fig. 1:**
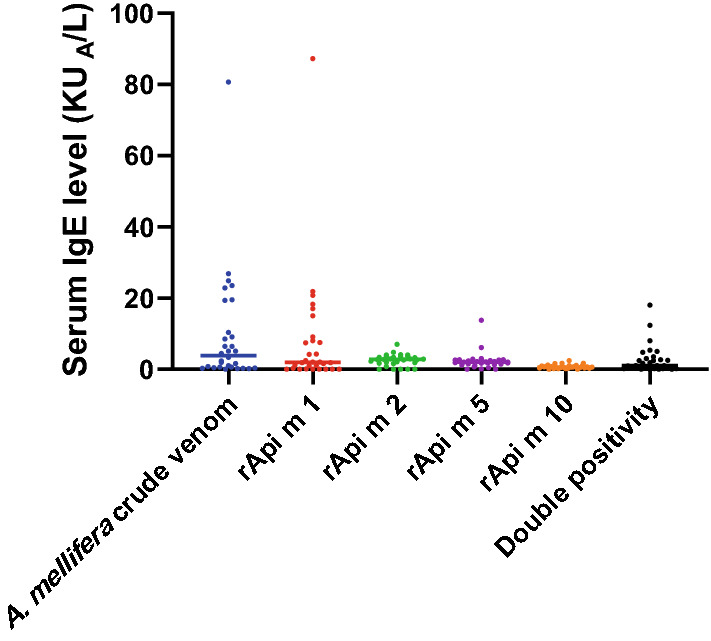
Evaluation of percentage positivity of IgE in Phadia ImmunoCAP; graph indicates IgE positivity to crude *A. mellifera* (immunologically similar to *A. dorsata)* venom, IgE positivity to venom components of *A. mellifera* in ImmunoCAP and “false” double-positivity (positivity to the venom of both honeybee and hornet)

**Fig. 2:**
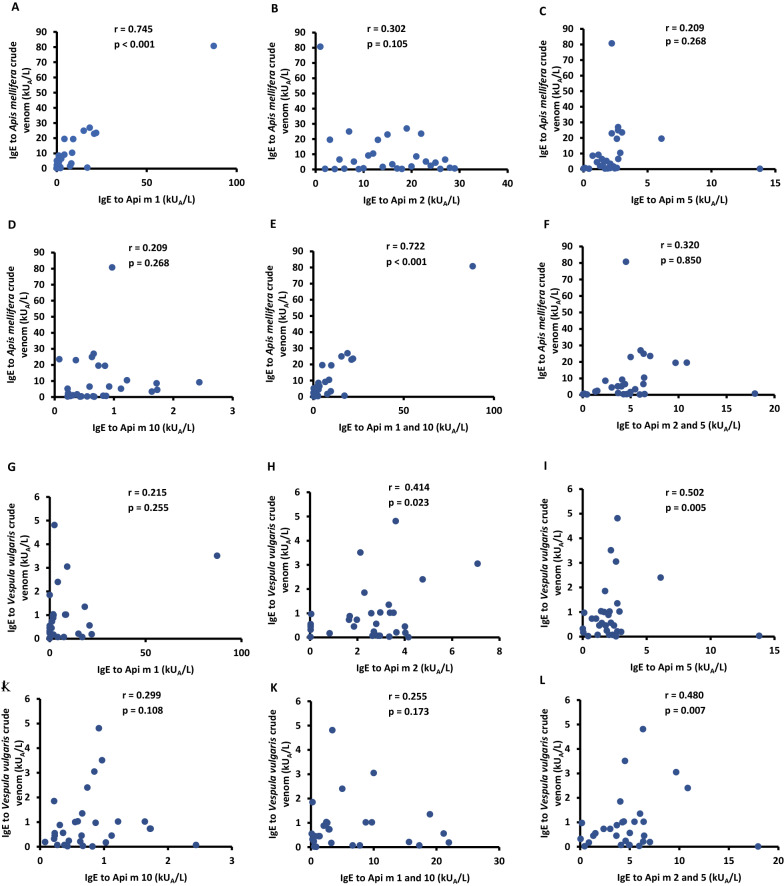
Correlation of IgE cross-reactivity to crude venom of *Apis mellifera* with that of recombinant allergens of *A. mellifera* venom; **A **rApi m 1, **B** rApi m 2, **C** rApi m 5, **D** rApi m 10, **E** rApi m 1 and 10 combined, **F** rApi m 2 and 5 combined. Correlation of IgE cross-reactivity to crude venom of *V. vulgaris* with that of recombinant allergens of *A. mellifera* venom; **G **rApi m 1, **H** rApi m 2, **I** rApi m 5, **J **rApi m 10, **K** rApi m 1 and 10 combined, **L** rApi m 2 and 5 combined. IgE to rApi m 1 and rApi m 1 and 10 combined showed a significant correlation with IgE to *A. mellifera* crude venom. IgE to Api m 2, Api m 5 and Api m 2 and 5 combined showed a significant correlation with IgE to *V. vulgaris* crude venom

Of the 30 patients who developed anaphylaxis to *A. dorsata* venom, 21 (70%), 25 (83%), 27 (90%) and 23 (76.6%) reacted to rApi m 1, rApi m 2, rApi m 5 and to rApi m 10 respectively, whereas 25 (83%) had positive sIgE to the crude venom of *A. mellifera* and 20 (66.3%) had positive sIgE to the crude venom of *V. vulgaris* venom (Fig. 1). Of the four recombinant allergens, IgE reactivity to rApi m 1 and 10 together had significant correlation (r = 0.722; p < 0.001) with the crude venom of *A. mellifera* (Western honeybee) and higher positivity rate of 90% (27/ 30). IgE reactivity to rApi m 2 or 5 had significant correlations of r = 0.414; p = 0.02 and r = 0.502; p = 0.005 respectively with *V. vulgaris* crude venom and rApi m 1 and 10 combine had lower correlation with the wasp venom (Fig. 2). No significant association was observed with IgE quantity and severity of the patients.

### Passive immune basophil activation in response to* A. dorsata* venom

**Fig. 3:**
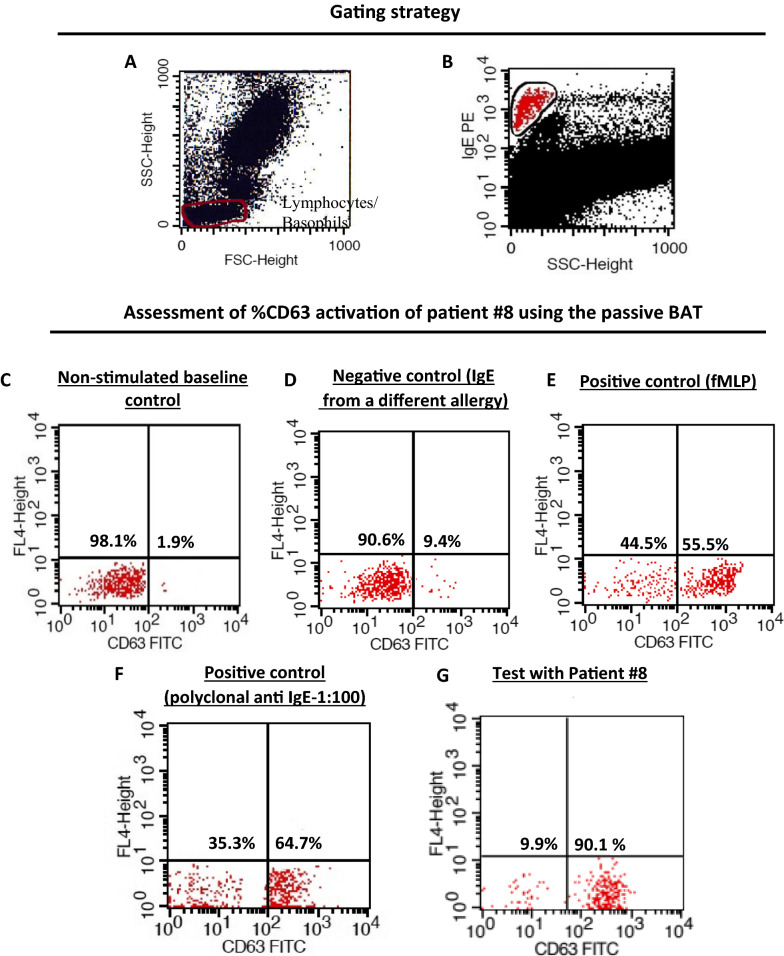
Upregulation and expression of CD63 in passively immune donor basophils by *A. dorsata* venom and evaluation of the percentage positivity of one patient; **A** gating of lymphocytes; **B** high IgE^pos^ population from the lymphocyte gate selected; **C** non-stimulated baseline control; **D** native control from an atopic non-Hymenoptera allergic individual; **E** positive control (fMLP); **F** positive control (polyclonal anti-IgE); **G** test with patient#8 using 1 µg/mL *A. dorsata* venom

**Fig. 4:**
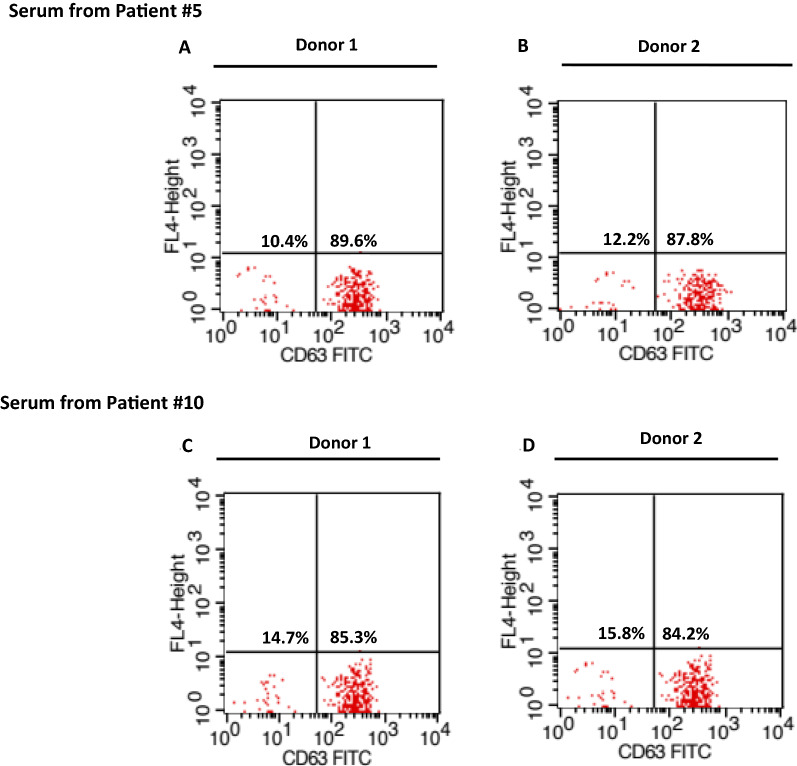
Evaluation of donor dependency of the test; **A** %CD63 activation of basophils from donor 1 incubated with sera from patient #5; **B** %CD63 activation of basophils from two donor 2 incubated with sera from patient #5; **C** %CD63 activation of basophils from two donor 1 incubated with sera from patient #10; **D** %CD63 activation of basophils from two donor 2 incubated with sera from patient #10 (all test were performed using 1 µg/mL of *A. dorsata* venom)

CD63 activation upon allergen stimulation of one test result (patient# 8) is shown in Fig. 3A–G. Polyclonal anti-IgE and fLMP used as a positive control gave a mean positivity of 65% and 55% of activated basophils (n = 3), respectively. Negative controls which were incubated only with stimulation buffer resulted in < 5% of CD63 expressing basophils. All seven negative controls (basophils with membrane-bound control IgE) resulted in < 10% basophil activation in the passive BAT in response to *A. dorsata* venom. All seven negative samples which had membrane-bound IgE to non-hymenopteran allergens (BSA) had very low (< 10%) basophil activation. The cutoff was set as 13% activation of basophils (mean + 3.3SD of negative controls). None of the double-positives in Phadia ImmunoCAP gave positive results with *V. affinis* venom in passive BAT. On repeating the test with a second donor, similar results were obtained as the first donor (Fig. 4).

**Fig. 5:**
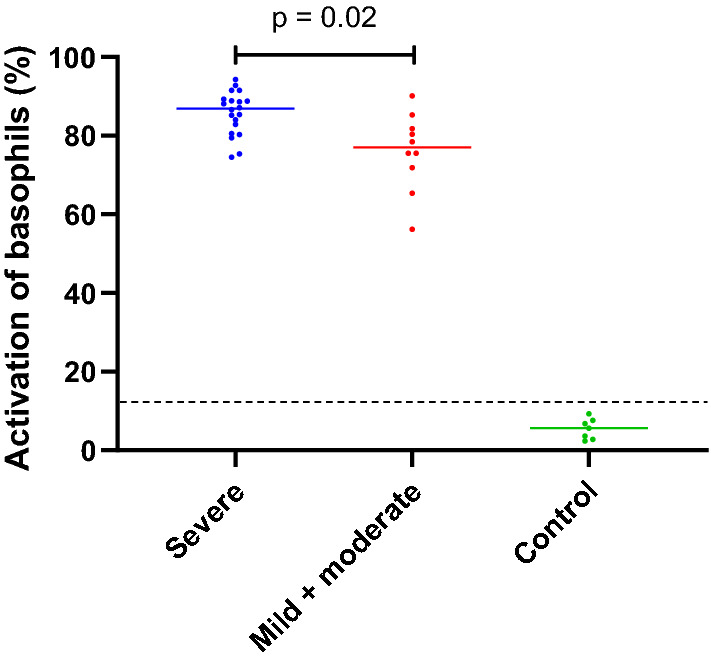
Comparison of percentage basophil activation in passive BAT and severity of the patients

All of the 30 (100%) patients with anaphylaxis to *A. dorsata* venom allergy were positive to *A. dorsata* venom in the passive BAT; 100% had over 50% activation, 96. 7% (29/ 30) had over 60% activation and 70% (21/ 30) had over 80% activation. Percentage activation of basophils in group 2 (n = 20: patients who had severe reactions) was significantly higher (p = 0.02: Fig. 5) compared to group 1 (n = 10: patients who had mild or moderate reactions).

## Discussion

This study was the first that evaluated the use of a panel of recombinant allergens of *A. mellifera *venom in diagnosis of *A. dorsata* venom allergic patients. We found a high rate of positivity to a combination of rApi m 1 and 10, compared to a lower rate of IgE positivity with rApi m 1 and 10 when done individually. A study from Germany and Sweden [21] showed a positivity rate of 86.8% with a combination of rApi m 1 and 10 in patients allergic to *A. mellifera*. The use of single components in the diagnosis of allergy to *A. mellifera* or *A. dorsata* showed lower positivity in previous studies as well. For example, the sensitivity was 72.2% and 61.8% for rApi m 1 or rApi m 10 respectively in *A. mellifera* allergy [21] and 44% for rApi m 1 in patients with anaphylaxis to *A. dorsata* in Thailand [7]. Therefore, individual use of rApi m 1 or 10 alone would not be sensitive enough in the diagnosis of *A. dorsata* venom allergy. There was a significant correlation of a combination of rApi m 1 and 10 with *A. mellifera* crude venom in our study, whereas there was no such correlation noted with *V. vulgaris* venom. rApi m 10 is clinically relevant but underrepresented in diagnostic venom extracts of *A. mellifera* [37, 38]. On the other hand, this molecule is present only in honeybee venom like rApi m 1. This indicates that combining the two genuine honeybee venom allergens, rApi m 1 and 10 would be sensitive in the diagnosis of *A. dorsata* venom allergy and could distinguish *A. dorsata* venom allergy from *V. affinis* venom allergy.

A high IgE positivity rate with rApi m 2 (hyaluronidase) and rApi m 5 (dipeptidyl peptidase IV) was seen in our study; however, we found significant correlations of rApi m 2 or 5 with *V. vulgaris* crude venom. Several previous reports from the West have also demonstrated the cross-reactivity of rApi m 2 and 5 with wasp venom. The cross-reactivity of rApi m 2 and 5 of *A. mellifera* and *V. vulgaris* may be due to the structural homology of these components [39]. We believe these homologous structures present in hyaluronidase and dipeptidyl peptidase IV of *A. dorsata* venom and *V. affinis*/ *V. vulgaris* venom may be the reason for all 20 patients who had positive IgE to *V. vulgaris* venom having IgE to rApi m 2 or 5. This indicates that the use of these two components to diagnose *A. dorsata* venom allergy has limited specificity and hence may impede the specific diagnosis of *A. dorsata* and *V. affinis* venom allergies.

The reactivity of donor basophils in patients with severe anaphylaxis compared to those with mild/moderate anaphylaxis was significantly high. As far as we are aware, this finding has not been evaluated with the conventional BAT for venom allergies and should be explored in further studies. Few studies on food allergies, particularly on peanut allergy [40, 41] have found a significantly high basophil reactivity in severely allergic patients with the conventional BAT. Studies on cat allergy and grass pollen allergy showed a reduced basophil activation after using omalizumab to reduce serum IgE [42, 43]. Interestingly, this reduced basophil activation was significantly correlated with clinical symptoms in an experimental allergen challenge [42, 43]. On the other hand, a correlation in severity and level of allergen-specific IgE levels in the serum has not been demonstrated [23, 44–46]. Moreover, one study showed low or undetectable serum IgE in 30% of patients [47] with venom allergy. Also, severe anaphylaxis can be possible in these patients with subsequent stings [44]. For such patients, passive BAT would be useful in diagnosis.

In contrast to the results from Phadia ImmunoCAP, the passive BAT showed a sensitivity of 100%. Reported sensitivity and specificity, in patients who had systemic allergic reactions (Muller grade II-IV), was 60–80% [27, 42], in conventional BAT. Reported sensitivity and specificity were within 85–100% [33–35] in the patients who had severe anaphylaxis (Muller grade III-IV reaction) to hymenopteran venom and the results are comparable with the present study.

In vitro testing by the Phadia ImmunoCAP indicated double-positivity with both bee and wasp venom in patients who reacted only to *A. dorsata* [20]. This may be due to the following reasons i) allergy to both species, ii) true cross-reactivity or iii) due to cross-reactive carbohydrate determinants (CCD) which are clinically irrelevant [16]. All our patients did not give a history of stings due to wasps/hornet, even though they showed positive IgE cross-reactivity to the venom of *V. vulgaris* by Phadia ImmunoCAP. However, the passive BAT showed a low activation of basophils for hornet venom. This indicates that it is useful in differentiating single positivity from false double positivity which is an important issue in the diagnosis of insect allergy.

Even though venom immunotherapy (VIT) is the only possible curative treatment for venom allergy [48], to date, there is no reliable in vitro test to monitor the success of VIT [48]. Hence, the sting challenge, though there are ethical issues in conducting the test, remains the only method that can evaluate the VIT [49] outcome. As CD63 expression of basophils only occurs in anaphylaxis [49], tolerance of culprit venom in allergic patients could be evaluated using conventional BAT replacing sting challenge [48]. The passive BAT may be developed as a functional assay to test the ability of IgE to induce activation of basophils.

The conventional BAT needs to be done within 4 h of venipuncture, whereas the passive BAT is less time-limited, as the patient’s serum rather than basophils are used. The passive BAT is more suitable than the conventional method, especially in Asia, where more stings occur in rural areas.

## Conclusion

A combination of rApi m 1 and 10 is sensitive in the diagnosis of *A dorsata* allergy. The passive BAT is highly sensitive in *A. dorsata* allergy diagnosis. Therefore, the diagnosis of *A. dorsata* venom allergy should be done initially with a combination of recombinant rApi m 1 and 10 and in instances of inconclusive results or double-positivity, the passive BAT should be included. The basophil reactivity in severe anaphylaxis compared to mild/moderate anaphylaxis was significantly higher. This finding should be explored in further studies.

## Data Availability

The datasets analyzed during the current study are available from the corresponding author on a reasonable request.

## Abbreviations

BSA: Bovine serum albumin
BAT: Basophil activation test
CCD: Cross-reactive carbohydrate determinants
CD: Cluster of differentiation
CRD: Cross reactive carbohydrate determinant
PBS: Phosphate buffered saline
PCH: Primary care hospitals
*TCH*: *Tertiary care hospital*
